# Crystallization screening: the influence of history on current practice

**DOI:** 10.1107/S2053230X1401262X

**Published:** 2014-06-27

**Authors:** Joseph R. Luft, Janet Newman, Edward H. Snell

**Affiliations:** a Hauptman–Woodward Medical Research Institute, 700 Ellicott Street, Buffalo, NY 14203, USA; b CSIRO Collaborative Crystallisation Centre, 343 Royal Parade, Parkville, VIC 3052, Australia; c Department of Structural Biology, SUNY Buffalo, 700 Ellicott Street, Buffalo, NY 14203, USA

**Keywords:** crystallization screening

## Abstract

While crystallization historically predates crystallography, it is a critical step for the crystallographic process. The rich history of crystallization and how that history influences current practices is described. The tremendous impact of crystallization screens on the field is discussed.

## Abstract 2

The rich history of crystallization and how that history influences current practices is described. The tremendous impact of crystallization screens on the field is discussed.

## 1. Introduction   

While one can argue about when structural biology was born, *e.g.* with the emergence of the X-ray structure of myoglobin in 1958, or the earlier structure of DNA, or perhaps when Bernal and Crowfoot showed that one could measure a diffraction pattern from a (hydrated) crystal of a protein in 1935, the importance of structural biology is without question. In the half century since the first myoglobin structure was published, 100 000 structures of biological macromolecules and macromolecular assemblies have been made available *via* the Protein Data Bank. Most of these have been determined by X-ray crystallography, a technique that relies on the work of many of the pioneers in diffraction, including von Laue and the Braggs, celebrated in this, the International Year of Crystallo­graphy. A fundamental requirement of the diffraction studies enabled by these early scientists is that the sample is crystalline, it is well ordered and of sufficient volume. The problem of producing crystalline samples for diffraction experiments is recognized as a major limiting factor of X-ray structure determination in structural biology. Recent advances in femtosecond X-ray protein nanocrystallography have made structural data collection from nanocrystals a reality (Chapman *et al.*, 2011 ▶) and have theoretically reduced the need for large single crystals. Although it is possible that in the future nanocrystals could become the standard for structure determination, currently the requirement for an X-ray free-electron laser (FEL) source to irradiate the crystals and the associated computational challenges in processing the resulting diffraction data means that this technique is not accessible to most investigators.

Protein crystals (used in the colloquial sense to encompass all biological macromolecules and assemblies) have been grown for well over 150 years. Giegé provides a comprehensive historical perspective on protein crystallization from the first observations in 1840 to the present day (Giegé, 2013 ▶). The first crystals were a serendipitous observation following the evaporation of earthworm blood under two glass slides (Hünefeld, 1840 ▶). Gradually more deliberate efforts followed, whereby the protein of interest was fractionated from its native source. In these early days crystals were not the goal of the experiments; crystallization was used as a purification process. The pioneering biochemists, having been trained in classical chemical purification, would have expected a crystalline solid on successful purification. Once the crystals were obtained, they were generally subjected to chemical analyses: % nitrogen, ash content, melt temperature *etc.* (Sumner, 1926 ▶) (difficult with protein crystals!). The purification process which yielded the early crystals would have relied on cycles of extraction (ethanol or acetone extraction), salt (ammonium sulfate) precipitation and precipitation *via* pH manipulation or temperature cycling. The proteins that survived these relatively harsh purification techniques might be expected to crystallize, as they would have necessarily been very stable.

We would hardly recognize these crystal-growing laboratories as being places equipped to do biochemistry, as many of the chemical, physical and analytical tools which we take for granted simply did not exist. SDS–PAGE analysis, for example, was developed over a century after the first protein crystals were noted (Summers *et al.*, 1965 ▶). Similarly, HEPES buffer and other similar buffers were first synthesized and characterized by Good and coworkers in 1966 (Good *et al.*, 1966 ▶); prior to this, the choice of appropriate buffers at neutral pH was very limited indeed. Practically, micropipettes with dispos­able tips were first available in the 1960s; prior to this, one used mouth-pipetting with glass capillaries (minimum volume 5 µl). Perhaps a telling example of the times is from the purification of jack bean urease by Sumner (1946 ▶), where extracts were cooled by leaving them on the windowsill overnight and then hoping for cold weather in lieu of a more controlled low-temperature environment. The refrigerator, which is a more recent version of the ‘ice chest’, is essential in today’s laboratory.

The ingenuity and techniques that were available to the early biochemists can sometimes still be glimpsed through the techniques in use today. Sumner, rather perceptively, describes a number of other characteristics of the jack bean urease protein and crystals which are worth noting (Sumner, 1926 ▶): the protein activity was quantitatively less from dilute protein solutions than from concentrated ones, which was attributed to dilute solutions of the protein being unstable, and concentrated solutions (if kept cold) maintained activity. Sumner also noted that whereas freshly prepared crystals dissolve readily in water, old crystals are insoluble and cannot be rescued by re-crystallization. The information that protein should be stored as concentrated as possible and that protein crystals degrade over time is as relevant today as when first published in 1926.

Today, in the majority of cases, the primary goal for growing protein crystals is for X-ray structure determination; extensive efforts have been invested in this process. There are many things to consider when growing protein crystals: the protein sample itself, the purity, the solubility and the stability are amongst the key considerations. The need for protein purity is captured by Berridge, who was investigating the purification and crystallization of rennin,

though crystalline form is not of itself complete and final evidence of either purity or true crystallinity, is it a matter of experience that unpurified enzymes cannot be crystallized and that quite small quantities of some impurities prevent crystallization

(Berridge, 1945 ▶). While there are certainly exceptions in the literature of proteins that crystallize from an impure state, for example from egg whites (Osborne & Campbell, 1900 ▶), the best approach for successful and reproducible crystallization is to begin with a consistently purified, soluble and stable protein formulation. The protein is the most important variable in crystallization (Dale *et al.*, 2003 ▶). This important crystallization variable, the protein and its formulation, can be controlled by the investigator, and should always be considered, first and foremost, before undertaking crystallization screening experiments. Furthermore, the protein itself can be altered by protein modification and formulation; such modifications can affect the stability and solubility of the protein and can dramatically increase the probability of crystallization. Cofactors, ligands and metals, all of which may bind to a the active site of a protein and stabilize it, are a particularly relevant class of chemicals for protein formulation.

Given pure protein, the next step is to understand its solubility. Prior to the widespread use of automation and commercially available crystallization screens, characterizing the solubility of a protein was a step that was typically performed before crystallization was attempted. This process was fruitful; it provided a knowledge-based foundation upon which to select the chemical conditions for crystallization, especially if only limited sample was available. The classical crystallization techniques used significantly larger quantities of protein than are commonly used today; even as recently as the 1980s crystallization drop volumes were on the scale of 5–10 µl, whereas today, using robotics, the majority of laboratories are screening at submicrolitre volumes. Probably the most common contemporary approach to crystallization is to purify a protein and then to immediately set up commercial crystallization screens. The benefit of this approach is that the protein is freshly prepared as it undergoes crystallization trials. The use of automated crystallization systems means that large numbers of low-volume trials can be set up rapidly; the diverse chemicals in the commercial cocktails can promote protein crystallization with little time for degradation. A potential drawback to this approach is that the protein has not been pre-formulated for solubility or stability. If degradation or amorphous aggregation occurs this can prevent crystallization or decrease the probability of being able to reproduce the results. Finally, and perhaps most importantly, it generally means that the crystallization will commence with little foreknowledge of the solubility behaviour of the protein. This behaviour informs crystallization: it tells the investigator where to search and where not to search for crystals. This is important considering the sizable multiparametric space that will be sampled to determine initial crystallization conditions.

Maximizing protein solubility and stability prior to commencing with crystallization screening will increase the number of crystalline outcomes (Jancarik *et al.*, 2004 ▶; Izaac *et al.*, 2006 ▶). The protein solution has to be sufficiently concentrated before crystallization so that supersaturation can be achieved during the trials and the protein has to be stable enough to remain correctly folded during the crystallization experiments. The current incarnations of solubility testing are generally modern extensions of the classical methods used to characterize protein precipitation points prior to crystallization screening (McPherson, 1976*b* ▶). The optimum solubility approach reported by Jancarik *et al.* (2004 ▶) is designed to identify the best buffer for protein stability. This is based first upon a lack of visible precipitation, followed by dynamic light-scattering analysis of the clear drops to verify that the protein is soluble and monodisperse prior to setting up crystallization screens. Another approach for protein formulation begins with flocculent protein precipitate, formed by dialyzing the protein against deionized water (Collins *et al.*, 2004 ▶) or through the addition of PEG 8000 (Izaac *et al.*, 2006 ▶), and then uses a series of solutions with varying salt, buffer and pH to fractionate the protein between precipitated and soluble states, thus measuring the solubility of the protein. Crystallization results can also be used for a *post mortem* analysis of protein solubility. Clear drops can be analyzed for chemical trends that relate to the relative solubility of the protein to identify potentially useful chemicals for protein formulation prior to the next round of crystallization screening (Collins *et al.*, 2005 ▶; Snell *et al.*, 2008 ▶).

A more recent technique that tests protein stability is differential scanning fluorimetry (DSF). In this technique, a hydrophobicity-sensitive dye (most often SYPRO Orange) fluoresces in a hydrophobic environment, while the fluorescence of the dye is quenched in an aqueous environment. The protein is heated and as it unfolds the dye can bind to the exposed hydrophobic core, giving a fluorescent signal. By monitoring this fluorescence, one can obtain a reasonable estimation of the melting temperature, *T*_m_, of the protein. Some studies have shown that a high *T*_m_ as measured by this technique bodes well for crystallization (Dupeux *et al.*, 2011 ▶), while in others the correlation is not as clear (Price *et al.*, 2009 ▶). The technique is performed in microplates and can rapidly probe the stability of a protein in many different chemical environments. If an individual protein is formulated in a chemical environment where it has a higher *T*_m_ value, this typically indicates that some component of that environment reduces the conformational flexibility of the protein, providing a more rigid structure that will have an increased likelihood of crystallization (Ericsson *et al.*, 2006 ▶). This method is particularly well suited to identify metals, cofactors and ligands that can promote intramolecular interactions to stabilize a particular conformation of a protein (Niesen *et al.*, 2007 ▶). DSF data should always be verified with dynamic light scattering, or a similar technique, to make certain that the increase in *T*_m_ value is not owing to protein aggregation

Once the protein has been prepared in an optimal buffer, crystallization trials can move forward. Some consideration should be given to batch-to-batch variation in protein preparations. If different batches of protein are prepared, attention should be paid to characterization of the protein to decrease the likelihood of encountering irreproducible results when translating from screening for initial crystallization conditions and eventual optimization of the crystals. Another consideration is that when super-expressers are encountered, or when very large batch preparations are possible, where a single, large lot of protein can be prepared, then the stability during storage needs to be evaluated. This can be accomplished by storing aliquots of the protein at different temperatures and then periodically assaying them to determine storage temperatures at which the protein remains viable. A few generalizations are to avoid lyophilization, and when freezing or thawing a protein sample to perform this rapidly (Deng *et al.*, 2004 ▶).

**Figure 1:**
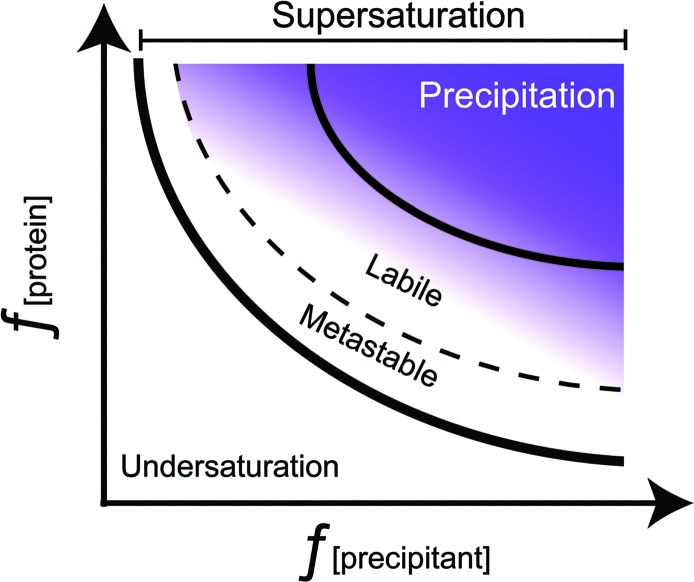
A simplified phase diagram for the crystallization of proteins. The phase diagram shows a concentration of protein *versus* a concentration of precipitant. The precipitant could be any chemical or physical variable that affects protein solubility. The undersaturated region is both kinetically and thermodynamically incapable of supporting crystal nucleation or growth. The thick boundary between undersaturation and the metastable region represents the saturation point of the protein. This is the endpoint after full equilibration of an experiment that produces a crystal. At saturation the crystal is in a state of dynamic equilibrium with the surrounding solution, which will always contain some protein. This saturation boundary has been measured in the laboratory for a small number of proteins; a selection of these are named in §[Sec sec1]1. The supersaturated regions are shown above the saturation boundary. The metastable zone is thermodynamically, but not kinetically, able to support spontaneous homogeneous nucleation events. The solution will remain clear. If a nucleant is introduced into a metastable solution, it can support growth of the crystal. The next highest level of supersaturation, the labile zone, is sufficiently supersaturated for spontaneous homogeneous nucleation. If the experiment is closer to the metastable zone, fewer nucleation events are likely to occur before entering the metastable zone. If the experiment is closer to the precipitation zone then a greater number of nucleation events are likely. The precipitation zone is many times supersaturated with respect to crystallization. Boundaries are shown between the metastable and labile zones, when in fact these boundaries only represent probabilities and, owing to the stochastic nature of the process, there can be overlap. Note that while only two axes are shown, multiple variables govern the solubility and the representation shown can be taken as only a slice through a complex multi-dimensional space.

To deliberately target crystallization it is useful to explore the mechanism of crystallization, as discussed in a recent review (McPherson & Gavira, 2014 ▶). At the basic level crystals are (simplistically) just an elegant form of ordered precipitation and occur when the supersaturation of the growth solution is sufficiently high after a random nucleation event occurs in an appropriate growth environment. Crystallization is best understood in the context of a phase diagram (Fig. 1 ▶). Determining an accurate phase diagram, with a single-crystal form (solid) and accurate protein concentration measurements in the surrounding solution (liquid) at true equilibrium between the solid and liquid phase, under ambient chemical and physical conditions is a nontrivial process which to date has been determined (with different levels of detail) for a small number of proteins which include bacteriorhodopsin (Talreja *et al.*, 2010 ▶), bovine pancreatic trypsin inhibitor (Veesler *et al.*, 2004 ▶), canavalin (Demattei & Feigelson, 1991 ▶), carboxypeptidase G_2_ (Saridakis *et al.*, 1994 ▶), chymotrypsinogen (Cacioppo *et al.*, 1991 ▶), collagenase (Carbonnaux *et al.*, 1995 ▶), concanavalin A (Mikol & Giegé, 1989 ▶), cytochrome *c* oxidase (Ataka *et al.*, 1992 ▶), glucose isomerase (Chayen *et al.*, 1988 ▶), haemoglobin (Green, 1931 ▶), insulin (Bergeron *et al.*, 2003 ▶), lysozyme (Ewing *et al.*, 1994 ▶), ovalbumin (Dumetz *et al.*, 2009 ▶), photosynthetic reaction centre (Gaucher *et al.*, 1997 ▶), ribonuclease A (Dumetz *et al.*, 2009 ▶), serum albumin (Rosenberger *et al.*, 1993 ▶), thaumatin (Asherie *et al.*, 2008 ▶) and xylose isomerase (Vuolanto *et al.*, 2003 ▶). Note that glucose isomerase and xylose isomerase are two names for the same protein, and although the solubility data were collected from two different species, *Arthrobacter* strain B3728 and *Streptomyces rubiginosus*, using differing methodologies and to different levels of granularity, the data are reasonably consistent. In cases where the phase diagram has not been fully determined, results from crystallization experiments can inform and help to sketch a rough phase diagram with limited solubility data to paint a logical progression for crystallization (Snell *et al.*, 2008 ▶; Asherie, 2004 ▶; Luft, Wolfley *et al.*, 2011 ▶).

The phase diagram in Fig. 1 ▶ is a simple representation of a complex, multi-variant process. This process is further complicated by the nature of the protein itself. Proteins are intrinsically unstable and the conditions which are used to engender supersaturation have to be chosen carefully to avoid denaturation. With this phase-diagram process in mind, we can explore how crystallization screens are designed to probe this chemical space.

With a basic understanding of the importance of crystallization in structural biology, the history of initial attempts at crystallization, the necessity for the best sample possible and an understanding of the phase diagram, we can begin to assess the influence of crystallization history on practice. We address only soluble proteins, as the important class of membrane proteins bring complexities unique to themselves and will be covered in a later article in this series. We describe a basic crystallization strategy and the influence of different methods on the trajectory of the experiment through phase space. We discuss the chemistry that drives this trajectory and how this is implemented efficiently with careful experimental design, leading to the many commercial screens that are in use today. Finally, we make observations on the process and attempt to show, for good or bad, how historical results have influenced today’s practices and what we might expect for the future.

## 2. Developing crystallization screens   

### 2.1. The first screening methods   

#### 2.1.1. Protein crystallization strategies prior to standardized screens   

Until late last century, the crystallization of biological macromolecules generally followed a well documented strategy that had been used by many crystallizers prior to the widespread success, availability and acceptance of pre-formulated crystallization screens. The approach (described below) is based upon and adapted from the publications of Gilliland (1988 ▶), McPherson (1976*b* ▶, 1982 ▶) and personal experience; it remains a completely valid approach and provides thoughtful guidelines for anyone attempting to determine initial crystallization conditions for a biological macromolecule.

(i) *Isolate the protein* using standard purification techniques to produce a pure, homogeneous and biologically active form of the protein. This step is critical for reproducing crystallization results. As noted above, while proteins can be crystallized from crude mixtures, this is not the best practice to obtain high-quality reproducible crystals for analysis by diffraction methods. Check that the protein is pure and that it is what you expect by as many techniques as you have available, but at a minimum SDS–PAGE analysis. Homogeneity should be considered in the context of the particular protein or protein complex being studied. If impurities do not resemble the sample then they may not be as detrimental as those cases where the target is microheterogeneous with contaminants closely resembling the crystallization target. Examples of those detrimental to crystallization heterogeneity would include protein–nucleic acid complexes where the nucleotides vary slightly in length, antibody–antigen complexes where the antigen is a homodimer and could lead to mixtures of Fab or antigen alone or in 2:2 or 2:1 complexes, and a protein that has partial occupancy of a ligand, a cofactor that dramatically alters the conformational state or stability or variations in post-translational modifications (such as phosphorylation), all of which produce structurally different states of a protein and yet would appear to be highly purified by SDS–PAGE analysis. It is critical to consider the source of contaminants to ensure that the biophysical methods used to detect them are appropriate to inform crystallization.
(ii) *Formulate and concentrate the protein* for crystallization in a buffer system in which it remains stable and soluble. A number of approaches can be used to formulate the protein in a crystallization-ready state. Typically, dialysis, ultrafiltration or size-exclusion chromatography is used to get the protein into a stable formulation where the pH and buffer type will vary depending on the activity, isoelectric point, solubility and stability of the protein. It is not possible to predict the formulation conditions under which the protein will be happiest, but there are some guidelines; for example, the pH of the formulation should be close to neutral and should avoid being too near the pI of the protein, as this is often a solubility minimum. If a high concentration (500 m*M* or greater) of salt or of glycerol (10% or greater) is required to keep the protein in solution this is an indication that the protein is potentially unstable, and rethinking the entire formulation or indeed protein construct may well be necessary. The point of crystallization trials is to perturb the protein in its storage formulation; thus, the formulation should be as dilute as possible to allow this perturbation to take place. The buffer should be in the concentration range 5–25 m*M*, weak enough that the addition of 10× concentrated buffer during crystallization attempts will significantly alter the solution pH. The salt concentration should ideally be below 200 m*M*. Other additives may be required for protein stability, including metal ions, cofactors or ligands, chelating agents and reducing agents, to name just a few of the chemical additives that have been used to stabilize protein formulations. A typical initial protein concentration range is from 5 to 15 mg ml^−1^, with some successful exceptions that are well outside this range of values. Crystals have been successfully grown from protein solutions containing protein from at as little as tenths of a milligram per milllitre up to hundreds of milligrams per milllitre, but generally 5–15 mg ml^−1^ is a reasonable starting concentration. For initial crystallization trials, the protein should be prepared in as concentrated a solution as it can be prepared in without showing signs of amorphous aggregation.
(iii) *Select chemical precipitants that have been reported frequently in the literature to produce protein crystals.* If the protein, or a member of a family of proteins, has previously been crystallized, initial experiments should focus on this class of chemicals. If the protein has not been crystallized, or fails to crystallize using these chemicals, the search should be expanded to include chemicals that have been most frequently reported in the literature as successful, including ammonium sulfate, 2-methyl-2,4-pentanediol and polyethylene glycol. These chemical recommendations are based on the first version of the Biological Macromolecular Crystallization Database (BMCD; Gilliland, 1988 ▶); a more recent version of the BMCD or other analyses of successful crystallization conditions from the Protein Data Bank (PDB; Tung & Gallagher, 2009 ▶; Peat *et al.*, 2005 ▶) should be consulted, but at first glance many of the chemical trends have remained remarkably consistent over time.
(iv) *Identification of protein precipitation points.*
  It is extremely useful if before actually setting up mother liquor for crystallization attempts, one acquires as good a feel for the precipitation behaviour of the macromolecule as possible
  (McPherson, 1976*b* ▶). This process, as described by McPherson, should be applied to a protein by titrating the protein drop at sequential pH values with one precipitant and then repeating this process at different temperatures. A depression slide, cover slips to prevent dehydration, a low-power microscope and the ability to add small aliquots of precipitant to a buffered drop of protein are the tools that are typically used to accomplish this task. A connection and understanding of the protein solubility prior to setting up crystallization experiments are the data required to develop a rational approach to crystallize a particular protein. These experiments should be performed at both room temperature and in a cold room to determine whether temperature affects the solubility of the protein. Obviously, if an effect is seen this adds an extra component to the strategy to make use of the effect.
(v) *Set up crystallization experiments spanning precipitation points.* Specific methods for sampling chemical space and setting up crystallization trials will be described in later sections. Regardless of the method used, be it batch, vapour diffusion or liquid diffusion, the concentrations of chemicals and the range of pH values used for the crystallization screen should encompass the ranges that have been predetermined from the solubility experiments that were used to establish the precipitation points of the protein. This approach, described as a ‘grid screen’ (Cox & Weber, 1988 ▶), enables a finer sampling of the protein solubility surrounding these precipitation points.
(vi) *Introduce chemical additives.* There are hundreds of chemically diverse additives that can be used to promote crystallization through different mechanisms. Many of these additives have been directly observed in crystal structures, stabilizing the protein or promoting lattice contacts, and can alter the physical chemistry of the solution to promote crystallization (McPherson & Cudney, 2006 ▶). These are added into the crystallization trials once the results of the trials set up in (v) have been determined.
(vi) *Select additional crystallization agents.* If crystallization attempts have failed, then expand the search to include additional precipitating agents or combinations of precipitating agents, *e.g.* PEG/salt, PEG/organic solvents, and repeat steps (iv)–(vi).

These steps are systematic and provide useful information about the protein and its response to different biochemical and biophysical conditions; however, they are time-consuming and somewhat tedious. One of the primary reasons for the almost instantaneous adoption of sparse-matrix screening using commercial screens is that it takes away the requirement to perform these painstaking but very useful experiments, but more particularly it takes away the need to invest time and thought into the crystallization experiment. A thoughtful experiment is always to be preferred, and in the long term is often the solution to more recalcitrant cases.

### 2.2. Crystallization methods   

#### 2.2.1. Definition of a crystallization method   

**Figure 2:**
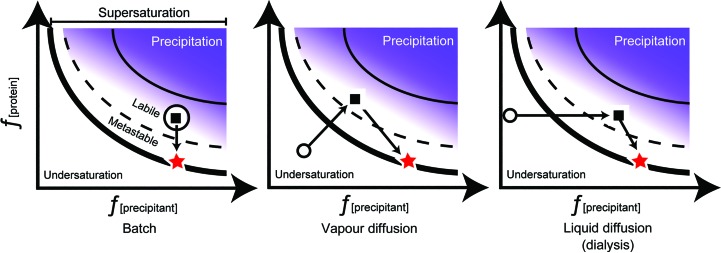
Idealized phase diagrams showing the trajectories of three different crystallization methods. From right to left, thermodynamic representations of batch, vapour-diffusion and liquid-diffusion (dialysis) experimental approaches to supersaturation, crystal formation and equilibrium (saturation). The open circle is the starting point of the experiment, the black square is the point of spontaneous homogeneous nucleation and the red star is the equilibrium point of the crystal. For batch experiments, the successful experiment is set up at labile supersaturation. A nucleation event takes place and protein in solution undergoes a phase change to the solid (crystalline) form. Equilibrium is reached when the protein in the surrounding solution reaches a state of saturation with the solid (crystal) phase. In the vapour-diffusion experiment, the initial drop conditions are undersaturated. As the drop dehydrates, typically through a dynamic equilibrium with the reservoir solution, the relative concentration of the protein and precipitant will steadily increase until the drop reaches a metastable state that will kinetically and thermodynamically support spontaneous homogeneous nucleation. The drop will typically further dehydrate as it equilibrates with the reservoir solution and the crystal will pass through the metastable zone; here it will grow to a larger size, but the solution will not be sufficiently supersaturated to support nucleation events. The drop reaches a saturation point when the drop and reservoir have equilibrated with respect to the vapour pressure of water, and the protein in the drop is in a dynamic equilibrium between the liquid and solid (crystalline) phase. The final example shows a liquid-diffusion experiment, in this case dialysis. The protein solution is held at a fixed volume. As precipitant passes through the semi-permeable dialysis membrane, the concentration of the precipitant will continue to increase while the protein concentration remains constant. When the solution reaches a metastable state then the protein will form a solid phase (crystalline). At this point, the concentration of the protein in the solution will decrease as protein transitions from a liquid to a solid phase. Saturation is reached when the solid and liquid phases have reached a state of dynamic equilibrium.

In the previous section, we very casually said ‘set up crystallization experiments’. Crystallization methods use physical and chemical means to induce supersaturation in a protein solution by manipulating the solution environment. There are a number of different techniques in use and the different methods will target specific variables (Luft & DeTitta, 2009 ▶). Conversely, the particular variables being investigated can guide the decision to select a crystallization method. The time required to set up a series of experiments must be considered, and the efficiency in terms of sample requirements and the number of variables screened in a given experiment should also be considered. Although it may take longer to set up an experiment, that experiment may in fact sample variables that another, easier method will not sample. Each method will have a unique trajectory through the phase diagram. Some, but not all methods, will have a set endpoint. The kinetics of equilibration, through dehydration of the protein-containing experiment drop or through liquid diffusion, will determine the rate at which supersaturation is obtained as well as the trajectory through the phase diagram and can often be passively controlled (Luft & DeTitta, 1997 ▶). Thus, the use of different crystallization methods is likely to produce different outcomes even when using identical stock solutions of protein and chemical cocktail. In summary, the crystallization method can be critical. There are three main categories of crystallization methods: batch, vapour-diffusion and liquid-diffusion. The crystallization method can be described as a convolution of the supersaturation kinetics driven by the crystallization cocktail and the supersaturation trajectory driven by the method:
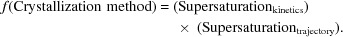
Fig. 2 ▶ illustrates how the method can influence the trajectory through phase space, again keeping in mind that the real situation can be far more complex owing to the multiple variables that can be involved.

In this paper, our focus is on the supersaturation thermodynamics, a process that is largely driven by the components of the crystallization screen used. Almost any variable that can be used to drive the supersaturation thermodynamics of a protein, without causing it to denature, has the potential to be exploited for crystallization. The key consideration for the crystallization methods chosen for screening is efficiency. For proteins, screening for crystallization is almost certainly a compromise between a complete multiparametric sampling of variables with the limitations of a small protein supply and is confounded by the complex variety and interactions of variables affecting crystallization. Crystallization screening is considered to be the most efficient method to sample the protein phase diagram (Dumetz *et al.*, 2007 ▶).

#### 2.2.2. Batch methods   

Batch experiments, in particular microbatch-under-oil (Chayen *et al.*, 1992 ▶) experiments, are conceptually simple: a protein solution is combined with a crystallization cocktail under oil; the oil is a barrier to dehydration of the experiment drop, but also acts as an interface that can affect crystallization. Batch experiments require similar volumes of sample and chemical cocktail solutions to set up the experiment, potentially making them extremely efficient from a cocktail perspective. The dehydration rate can be affected by making the oil barrier less or more water-permeable, for example by combining paraffin (less water-permeable) and silicone-based (more water-permeable) oils (D’Arcy *et al.*, 1996 ▶). The combination of paraffin and silicone oil in a 1:1 ratio, or even the use of 100% silicone oil, has been demonstrated to provide a greater number of crystallization hits than comparable paraffin-oil-only microbatch-under-oil crystallization screens (D’Arcy *et al.*, 2003 ▶). Experiments set up using solely paraffin oil will still dehydrate, albeit more slowly; water leaches through the plastic plates used for crystallization screening, which are typically somewhat water-permeable. Microbatch-under-oil experiments are especially compatible with temperature changes. They do not suffer from the condensation in the experiment well that can occur when transferring vapour-diffusion experiments from warmer to cooler temperatures.

#### 2.2.3. Vapour-diffusion methods   

Vapour-diffusion crystallization techniques such as the hanging-drop and sitting-drop methods are the most commonly used techniques for crystallization. A small droplet containing both protein and cocktail is dispensed onto a surface, often one that has been pre-treated so that surface wetting is minimized and a hemispherical droplet forms. The experiment droplet is then sealed in an airtight chamber with a reservoir solution. The drop undergoes a dynamic equilibration with the reservoir solution until the vapour pressure of any volatile species, typically water, over the experiment drop and the reservoir reach a state of equilibrium. While it is often the case that the reservoir solution is the same chemical cocktail that has been added to the protein solution, this is not a requirement. The purpose of the reservoir solution is to dehydrate the experiment drop and to set the endpoint for the dehydration. A variety of salt solutions have been used as a universal reservoir to increase the rate of dehydration, or to further dehydrate the experiment drop past the endpoint that would typically be achieved with the cocktail solution (Luft *et al.*, 1994 ▶; McPherson, 1992 ▶; Dunlop & Hazes, 2005 ▶; Newman, 2005 ▶). This can have the advantage of higher levels of supersaturation in the experiment drop; it can also lead to the unintentional formation of salt crystals. Perhaps it is appropriate to point out that the experimental methods designed to engender supersaturation in protein solutions can very often engender supersaturation and crystal growth of other components of the experimental system: the production of salt crystals is endemic in protein crystallization experiments. Some of these are very well understood: the very small solubility constants for magnesium phosphate and calcium sulfate almost guarantee that these will crystallize if given an opportunity. This happens (more often than not) when a phosphate buffer is used for protein purification and the resulting sample is set up in commercial sparse-matrix screens. Most of the common screens used for initial crystallization have magnesium in over 20% of the conditions; its presence is owing to the general effectiveness of magnesium for stabilizing intramolecular contacts to promote crystallization.

#### 2.2.4. Liquid-diffusion methods   

Liquid-diffusion techniques include microdialysis (Zeppezauer *et al.*, 1968 ▶; Lagerkvist *et al.*, 1972 ▶; Lee & Cudney, 2004 ▶), counter-diffusion (García-Ruiz, 2003 ▶) and free-interface diffusion (Salemme, 1972 ▶). Free-interface diffusion is generally based on a single precipitation event, whereas counter-diffusion exploits the difference in the speed of diffusion between protein molecules and small molecules, and is designed to generate multiple precipitation events at different levels of supersaturation. If a protein solution is carefully brought into contact with a solution containing a precipitating agent such as a salt in a manner which does not set up mixing by convection, the salt will move as a wave into the protein solution, while the protein molecules, being so much larger and thus so much slower to diffuse, essentially stay in the same place. There are a few well established ways of introducing a protein sample to a crystallization cocktail without convective mixing, with performing the experiment in zero gravity being one. More accessible techniques include using a very constrained geometry, such as a capillary with an internal diameter of 200 µm or less, or gelling one or both of the two components. These experiments trace a quite unique path through phase space and have the advantage of providing a gradient of concentrations of the faster moving components. Although a number of groups use this method almost exclusively and have shown it to be effective, it is not as widely used as the batch or vapour-diffusion techniques described above. This method is particularly suited to miniaturization in microfluidic chips, of which there are a number available commercially.

Dialysis methods are rarely used for crystallization screening, but are certainly worthy of mention; they trace a unique path through the phase diagram, holding the protein concentration constant until a phase transition takes place. The experiments are conceptually simple. A protein solution is placed within a container, and the container is sealed with a semi-porous dialysis membrane which has a molecular-weight cutoff (MWCO) that is small enough to prevent the protein molecules from escaping from the container. The container is placed within a larger reservoir solution and molecules below the MWCO of the membrane can then diffuse in, or out, of the protein solution to drive the system to supersaturation. Microdialysis methods have long been practiced (Zeppezauer *et al.*, 1968 ▶) and can be extraordinarily effective when a protein, such as the insecticidal δ-endotoxin CryIIB2, can be driven to supersaturation by reducing the concentration of a salt required for protein solubility (Cody *et al.*, 1992 ▶).

#### 2.2.5. Differences between methods   

There is obviously different parameter space being sampled by the different methods used, as noted in Fig. 2 ▶. These include very different kinetics of equilibration and solute concentrations at equilibrium/endpoints, distinguishing the microbatch-under-oil from the vapour-diffusion (and liquid-diffusion) methods (Luft, Wolfley *et al.*, 2011 ▶). There can also be more subtle differences, for example between air–water and air–oil interfacial phenomena (Maldonado-Valderrama *et al.*, 2005 ▶); these interfacial effects can affect crystallization. These variables contribute to the variation in results between methods, *e.g.* those that have been observed when studying, comparing and contrasting microbatch-under-oil with vapour-diffusion crystallization (Chayen, 1998 ▶). In general, comparative studies between modified microbatch-under-oil (D’Arcy *et al.*, 2003 ▶), where the experiment drops can dehydrate, and vapour-diffusion crystallization show that while there are some differences in the cocktails that produced crystallization hits when comparing the two methods, both methods are equally successful (D’Arcy *et al.*, 2004 ▶).

## 3. Crystallization chemistry   

### 3.1. General overview   

The process of supersaturation is driven by chemistry. All chemical agents that have been used to drive a protein to supersaturation have at least one common property: they will all act to promote protein–protein intermolecular interactions, leading to a phase change. These chemicals will act through different, for the most part well understood, mechanisms, dependent upon their chemical classification. Classes of crystallization agents include buffers, organic solvents, salts, polymers and small-molecule chemical additives.

#### 3.1.1. Buffering agents   

Altering the solution pH can be achieved using buffer solutions. The effect of the buffer is to change the surface charge distribution of the polyionic protein, which is likely to have an anisotropic charge distribution. The pH value where the protein has a net charge of zero (that is, where there are an equal number of positive and negative charges on the surface of the protein) is referred to as the isoelectric point or pI. Under conditions of low ionic strength, where pH_solution_ = pI_protein_, the protein has a higher probability of interacting with surrounding protein molecules because the positive and negative surface charges are likely to be ‘neutralized’ by interacting with other protein molecules, such that a positive patch on the surface of one protein molecule will contact a local negatively charged region on the surface of another protein molecule. Where pH_solution_ < pI_protein_ the protein will have a net negative charge; where pH_solution_ > pI_protein_ the protein will have a net positive charge. In the absence of other chemical species, this will create an environment where every protein molecule will have the same overall charge, and as like charges are repulsive the protein molecules will tend to move away from each other, which is seen as an increase in their relative solubility compared with a situation where pH_solution_ = pI_protein_. This makes pH a particularly important chemical variable for crystallization.

#### 3.1.2. Organic solvents   

Another class of chemical agents used to drive supersaturation are the organic solvents that can be used, among other physical chemical properties, to alter the dielectric constant of the solution, which in turn affects the amount of charge that is perceived on molecules. A lower dielectric constant typically equates to lower protein solubility. Organic solvents are most often used as additives, rather than as solo precipitating agents. They are typically volatile, which can make harvesting crystals a challenge. At higher concentrations, organic solvents will typically denature proteins.

#### 3.1.3. Salts   

Salts can act to shield charges between protein molecules and to form salt bridges that can promote favourable intermolecular interactions. Salts can also act by having a greater affinity for water molecules than the protein, forcing the proteins to interact through hydrophilic or hydrophobic interactions in the absence of available water molecules. Anions and cations follow a lyotropic series, the Hofmeister series, in which they are rated according to their effectiveness at dissolution of proteins. This series is affected by the pI of the protein and the pH of the solution (Kunz *et al.*, 2004 ▶). Chaotropic salts such as sodium bromide can interact with a protein and cause it to partially unfold, exposing interior hydrophilic residues to the solution to promote solubility.

#### 3.1.4. Polymers   

Polymers such as polyethylene glycol make water molecules unavailable to the protein through solvent-exclusion effects (Atha & Ingham, 1981 ▶), essentially trapping water molecules in regions to which the protein does not have access, rather than holding them in a higher affinity grasp as is the case with salts (Dumetz *et al.*, 2009 ▶).

#### 3.1.5. Additives   

Additives are a diverse class of agents; they can stabilize or alter the conformation of a protein, they can alter the physicochemical properties of the mother liquor to affect protein–solvent interactions and they can take part in reversible intermolecular interactions that promote crystallization (Larson *et al.*, 2008 ▶). Distinguishing between these two modes of action can be useful, as additives that engender increased protein stability may be appropriate to include during the purification process. The additive class includes small molecules that bind specifically to the surface of the protein and allow crystal contacts to be made between neighbouring protein molecules, commercialized as ‘Silver Bullets’: small molecules that could act to promote lattice interactions (McPherson & Cudney, 2006 ▶). One of the challenges faced by investigators attempting to analyze large numbers of chemically diverse additives was the combinatorial nature of the search for crystallization conditions. A successful simplification of this problem was devised by using a limited set of crystallization reagents and using combinations of the chemical additives in a single cocktail (McPherson & Cudney, 2006 ▶). The additives tested included organic salts and acids, biologically active molecules, peptides, amino acids and digests of macromolecules. Although biomacromolecules are fundamentally made up of the same chemistries (small numbers of amino acids, nucleic acids and sugars), as a group they are extraordinarily diverse and thus the additive class of molecules, which tend to make specific interactions with the protein, is large and difficult to summarize neatly.

Detergents can also be considered as additives. While detergents are commonly used for the crystallization of membrane proteins, the use of detergents at low concentrations as additives for soluble proteins has been shown in some cases to reduce nonspecific aggregation owing to hydrophobic interactions, improve reproducibility, increase the growth rate and increase the number of large single crystals (McPherson *et al.*, 1986 ▶; Cudney *et al.*, 1994 ▶). Glycerol, at a concentration sufficient to form an amorphous glass at 100 K, can be added as a cryoprotectant; this been added to the Jancarik and Kim sparse-matrix screen to produce a cryo-ready version of this classic screen (Garman & Mitchell, 1996 ▶). Glycerol and other polyols can also be used as protein structure-stabilizing agents (Sousa, 1995 ▶). Ionic liquids are an interesting class of additives. The potential mechanisms by which they effect crystallization are numerous, but they have proven to be effective in a number of cases (Pusey *et al.*, 2007 ▶).

## 4. Experimental design   

### 4.1. Overview   

The chemical and physical parameter space that a protein can comfortably occupy is vast. An effective strategy is needed to search for crystallization conditions. Discovering initial crystallization conditions, assuming that the protein will crystallize, is a search problem (Kingston *et al.*, 1994 ▶). A modest initial set of screening conditions set up in a sequential manner and learning from the initial trials where best to focus the search in subsequent experiments is desirable from the perspective of sample efficiency, but necessarily requires time for the first series of experiments to produce a result and be analyzed prior to the design and set up of the second series. This approach is further confounded by the unfortunate tendency of protein samples to denature over time.

### 4.2. Parameter space   

Parameters for crystallization screening can include continuous variables, such as concentration, pH and temperature, and discrete variables, such as a specific chemical type, independent of its concentration. Practically, the continuous variables may have to be considered as discrete: while temperature is clearly a continuum, there may however only be a very limited number of temperatures available at which to incubate crystallization trials.

#### 4.2.1. Sampling methodologies   

**Figure 3:**
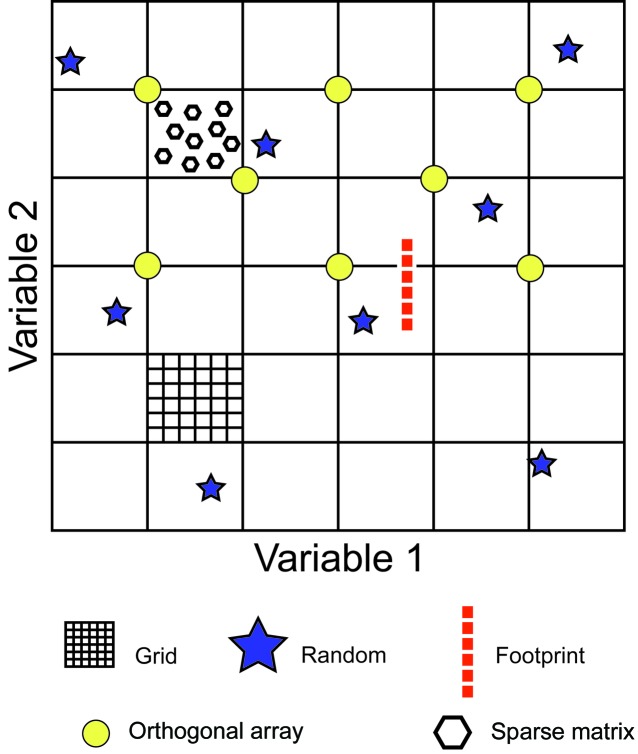
Sampling of variables in two dimensions. Random sampling (blue stars) is considered to be among the best approaches for crystallization success. While random sampling covers a broad range of parameter space, sparse-matrix sampling (white hexagons) is a random screen that focuses on variables known to have had past success. An orthogonal array (yellow circles) is a symmetric sampling of random space. Footprint screen (orange squares) sampling begins by incrementally searching in a narrow range of variables. Adapted from Segelke (2001 ▶).

It is simply impractical to set up every crystallization experiment that could be conceived of for a given protein; there are too many variables and there would never be enough time and protein to make this even a remote possibility. Regardless of the particular chemical cocktails that we set up to identify initial crystallization conditions, it is going to be a sampling problem. Screens can be classified in terms of their approach to sampling chemical space (Fig. 3 ▶), with multiple potential solutions to the problem. The space and fidelity of sampling depends on both the approach and the number of experiments. Random screens are considered to be a very effective strategy (Segelke, 2001 ▶). Based upon an analysis of the probability of success for crystallization from random sampling of crystallization conditions, ∼300 experiments would be a thorough screen (Segelke, 2001 ▶). That said, within the High-Throughput Crystallization Laboratory at the Hauptman–Woodward Medical Research Institute we have observed many cases where a protein will crystallize in only one cocktail from a 1536-cocktail microbatch screen (Luft, Snell *et al.*, 2011 ▶). Where more than one condition produces a crystal or an identifiable result within the phase space of the protein, the additional information provides data that expand the chemical knowledge of the solubility behaviour of a protein to rationally guide sequential experiments (Snell *et al.*, 2008 ▶). The nature of the experiment is also important in sampling. In batch experiments chemical space is sampled as a discrete rather than a continuous variable, whereas in diffusion-based experiments a dynamic component is included. The search problem is confounded by the stochastic or random nature of nucleation: it cannot be assumed that just because a crystal did not form in a particular experiment that a crystal cannot form under these conditions (Newman *et al.*, 2007 ▶).

One of the most widely used approaches to design crystallization screens dates back to the work of Carter and Carter, who described the concept of combining the two principles of randomization and balance, conceptualized through an incomplete factorial design, as a strategy first used to develop a crystallization screen for *Bacillus stearothermophilus* tryptophan-tRNA synthetase (Carter & Carter, 1979 ▶). In this approach, a screen was designed that could be used to effectively identify variables significantly correlated with crystal quality and that provided greater insight into intelligent iterative crystallization screen design than the standard practice of controlled single-factor and full-factorial screens. The approach of Carter and Carter led to the development of sparse-matrix screens; these are essentially random screens that have been biased toward chemicals that have previously been used to crystallize a protein. These ‘directed’ random screens are the most efficient way to identify initial crystallization conditions (Segelke, 2001 ▶).

The use of orthogonal arrays to design initial crystallization screens has also been described (Kingston *et al.*, 1994 ▶). This approach is based upon the selection of a nearly symmetric subset of a full-factorial design with a uniform distribution of points. The advantages of orthogonal arrays include having a tractable number of experiments in which to explore chemical space in a systematic manner, providing a logical foundation for subsequent analyses and further experimentation.

Finally, there is the grid-sampling approach which has the advantages of being simple and direct (Cox & Weber, 1988 ▶). A grid screen will typically use two components: a precipitating agent at a series of coarse concentration increments and a second pH-buffering component which is also coarsely sampled. While it does not screen a wide region of chemical space, this type of screen can be extremely effective and especially valuable when the protein is in very limited supply. Grid screens provide readily interpretable solubility information and highlight regions where finer successive screens should be undertaken. While limited in chemical scope, the effectiveness of proven champions of crystallization, such as PEG or ammonium sulfate, sampled against a range of pH values can be an effective crystallization strategy.

#### 4.2.2. Drop volume   

Using smaller drop volumes allows a greater number of screening experiments to be set up using the same volume of protein. The advantages include an opportunity to expand and apply crystallographic methods to include biological macromolecules that are nearly impossible to supply in amounts sufficient for more traditional approaches. However, from a practical standpoint, decreasing the drop volume decreases both homogeneous and heterogeneous nucleation rates; for homogeneous nucleation of tetragonal lysozyme crystals there is a linear relationship to drop volume, experimentally determined to be of the order of one nucleation event per ∼10^−1^ mm^3^ per 24 h (Bodenstaff *et al.*, 2002 ▶). Based upon this value, to achieve roughly the same nucleation rate on scaling up from a 400 nl screening experiment to a 4 µl experiment requires an ∼1000-fold decrease in the level of supersaturation. This partially explains the well known and very frustrating problems of ‘scale-up’. In practice, this means that rare nucleation events leading to diffraction-quality crystals may be less likely to be observed in smaller drops. The stochastic nature of nucleation, and its dependence on drop volume, should not be confused with the size (volume) of any eventual crystals, which will also be governed by drop size; more specifically, the latter will be governed by the amount of material available for inclusion in the growing crystals.

### 4.3. The first crystallization ‘kit’   

In 1991, crystallization changed when Jancarik and Kim developed a set of ‘reasonable’-looking crystallization conditions based on the chemicals that had been successful in previous crystallization experiments (Jancarik & Kim, 1991 ▶). They called this collection of likely conditions a ‘sparse-matrix’ sampling of crystallization space. At the time, the PDB contained <500 structures, so the basis for these conditions was not extensive. It was the genius of Jamula Jancarik to recombine the chemical factors she identified into a set of conditions that continues to dominate crystallization screening to this day. The sparse-matrix screen developed is a set of 50 chemical solutions that are heavily biased towards published crystallization conditions and recognize the influence of the incomplete factorial approach (Carter & Carter, 1979 ▶). This screen samples five pH values with associated buffers, four precipitating agents and eight salt additives known to have been successful for the crystallization of proteins. It is a chemically broad search with very coarse sampling. The impact that this screen had on protein crystallization is tremendous and cannot be adequately conveyed by the >2000 citations that the publication has thus far received. Not only has it been very effective at crystallizing proteins, as seen by the fact that it is still one of the most widely used screens today, even in a crowded field of over 200 commercially available screens (Newman *et al.*, 2013 ▶), but also it lowered the barrier to crystallization. The sparse-matrix screen was a constant, making it well suited for automation. It was a means for an absolute novice to start down a path to identify crystallization conditions. It was now possible to quickly test a protein for crystallization using very little sample, time and prior expertise. Of course the ‘little time’ is relative; to formulate each of the 50 solutions in a laboratory was a considerable undertaking. An indication of how exciting this development was is seen in the rapid translation of the publication into the first commercially available screen within months. Hampton Research (Aliso Viejo, California, USA) produced a commercial version of the Jancarik and Kim screen as ‘Crystal Screen’ in the same year as its publication. Commercial availability was an important event that led to the widespread development and propagation of crystallization kits. The only feature of the initial Jancarik and Kim screen that has not stood the test of time was their selection of 50 conditions for the screen: conditions 49 and 50 of the original Jancarik and Kim screen are little used and the screen is combined with another 48-cocktail screen (often Crystal Screen 2 from Hampton Research) to conveniently fill all 96 positions of a microplate. Based upon developing practices, glycerol was added in concentrations appropriate to act as a cryoprotectant, making every cocktail in the screen cryo-ready (Garman & Mitchell, 1996 ▶).

### 4.4. The development of crystallization strategies through further kit design   

#### 4.4.1. Sparse matrix   

The introduction of the sparse-matrix screen as a general tool for the crystallization of soluble proteins and its rapid adoption by the field was followed, logically, by a series of screens that specifically targeted different classes of biological macromolecules that were based upon the sparse-matrix approach. Crystallization assays that targeted ribozymes and small RNA motifs (Doudna *et al.*, 1993 ▶) and hammerhead RNAs (Scott *et al.*, 1995 ▶) suitable for the crystallization of both RNAs and RNA–protein complexes were developed. These screens have similar components, as would be expected; however, the screen developed for the crystallization of hammerhead RNAs relies more heavily on the use of PEG of varying molecular weights coupled with monovalent salts as precipitants. Like Crystal Screen, these screens consist of combinations of chemicals which were found in conditions used to crystallize RNA. A similarly focused screen used a 24-cocktail matrix for the crystallization of DNA and RNA oligomers (Berger *et al.*, 1996 ▶) with MPD (2-methyl-2,4-pentanediol) as the only precipitating agent. Another example of the use of accumulated crystallization data from the PDB (Berman *et al.*, 2000 ▶) and BMCD (Gilliland *et al.*, 1994 ▶) was the development of a crystallization screen specifically designed for the crystallization of protein–protein complexes based upon a coarse categorization of precipitants (PEG, ammonium sulfate, other salts and organic solvents) that successfully crystallized protein–protein complexes, followed by a finer search to identify the most effective types of PEG, range of precipitant concentrations, buffer, pH and lower concentration salts (Radaev & Sun, 2002 ▶). They grouped together the known protein–protein complex crystallization conditions and used a cluster analysis to generate the 48 most probable cocktails for the crystallization of a protein–protein complex, which included 39 PEG conditions and nine ammonium sulfate and other salt conditions with pH values between 6.0 and 8.5.

Five component categories (buffer/pH, organic precipitating agents, salt, divalent cations and additives) were selected as ingredients for a statistical experimental design for protein crystallization screening (Tran *et al.*, 2004 ▶). This screen contains 48 cocktails, with the choice of chemicals based upon those most frequently reported in the BMCD and in publications. The advantages of the statistical design included a comparable success rate to other screens with a smaller number of chemicals, with a more straightforward path towards optimization than a random screen owing to the repetition of specific chemicals within the screen (Tran *et al.*, 2004 ▶). More recent examples of this same approach of data mining and creation of screens to encapsulate the results can be found in the Morpheus screen (Gorrec, 2009 ▶) and the MemGold screens (Newstead *et al.*, 2008 ▶; Parker & Newstead, 2012 ▶).

#### 4.4.2. Footprint screening   

The ‘footprint screen’ (Stura *et al.*, 1992 ▶) is designed to coarsely sample the protein precipitant solubility curve at three pH values using two classes of precipitating agents, three PEGs and three salts, at four concentrations. This is a modernized version of the classical approach to determine the protein solubility under a limited set of chemical conditions prior to initiating complex crystallization screens. This screen efficiently compares the solubility behaviour of macromolecules, complexes and aliquots from different purification protocols and informs the investigator to select preferred precipitants for the further investigation of crystallization conditions. This requires very small amounts of protein and through this rapid assessment of the solubility behaviour enables one to rationally direct sequential crystallization experiments: ‘reverse screening’ (Stura *et al.*, 1994 ▶).

#### 4.4.3. Grid screening   

The use of successive automated grid searches (Cox & Weber, 1988 ▶) was an approach that was developed into commercially available grid screens. This approach does not focus on chemical diversity so much as a relatively fine sampling of the concentration of a particularly effective crystallizing agent *versus* pH. In their original design, a 4 × 4 broad grid screen initially surveys the response of the protein to four values of pH (2.0 ≤ pH ≤ 8.0) and four precipitating agent concentrations. Three commonly used precipitating agents were selected for the initial screen, which included ammonium sulfate, PEG 8000 and a PEG/salt mixture. The buffer for the initial screen, citric acid–sodium phosphate buffer, was selected to cover a broad pH range. This initial search was narrowed in successive screens to produce larger crystals. Additional variables were additives, including salts and detergents, which were added to these grid screens at a single concentration. Temperature was also investigated by placing crystallization trays at 277, 291 or 303 K. It should also be noted that Cox and Weber were conscious of the requirements of the protein for stabilization and included specific additives to address this prior to crystallization screening.

#### 4.4.4. Knowledge-based screening   

Most crystallization screens are designed to accommodate the widely varying physical-chemical properties of proteins. For instance, most screens will cover a wide range of pH values. Investigators will typically apply a commercial screen to their proteins using all of the cocktails in the screen, even when they have prior knowledge that a particular protein may be chemically incompatible with some of the cocktail conditions. The concept of a modular approach, in which specific chemical variables in a crystallization screen are tailored to the physical-chemical characteristics of the protein, was proposed by Kingston *et al.* (1994 ▶). Investigators who are undertaking crystallization screening will only rarely dissect commercial screens to select cocktails known to be chemically compatible with their protein. The efficiency of setting up the standard crystallization screens often supersedes more sample-efficient approaches which, while they require more time to initially construct, will likely be a less time-consuming approach in the long run for more challenging crystallization targets.

A screen that was not focused on a single class of proteins, but was more of a protein-centric screen, was developed and referred to as the ‘Clear Strategy Screen’ (Brzozowski & Walton, 2001 ▶). This screen takes into account five key observations to help minimize the number of cocktails in initial crystallization screens. These observations as outlined include that there are common trends in the crystallization of chemically or structurally similar macromolecules (Hennessy *et al.*, 2000 ▶), that only a few conditions may be required to crystallize a high percentage of well characterized proteins (Kimber *et al.*, 2003 ▶), that in most cases crystallization conditions are relatively simple chemically and that folding homogeneity is the basic prerequisite for crystallization success. The pH of the limited set of PEG and salt cocktails is set by the user based upon prior knowledge: experimental characterization of the physical-chemical properties of the protein. This approach uses specific data regarding the stability and aggregation of the protein at different pH values to perform a final formulation of the screen. The formulation of the cocktails also takes into account cryoprotection of crystals by including PEG 1000 and PEG 550 MME in cocktails containing PEG 8000 and PEG 20 000 to enable more direct cryopreservation of any resulting crystals. The goal of the authors who developed the Clear Strategy Screen was to highlight its simplicity and efficiency with the hope of instigating

more rational logical and flexible approaches to crystallize macromolecules

(Brzozowski & Walton, 2001 ▶).

### 4.5. Chemically focused screens   

As well as screens developed through data mining, there were some that were developed to encapsulate the concept of limited screening using a set of pre-formed conditions, but where the conditions were based around prior knowledge. For example, it was known that complete antibodies tended to crystallize in low ionic strength conditions, so a screen consisting of such conditions was created (Harris *et al.*, 1995 ▶). Similarly, the precipitant synergy screen designed at Columbia University captured the belief that certain chemicals work better in combination than in isolation, and resulted in the commercially available ‘Precipitant Synergy’ screen, which uses combinations of chemically distinct precipitant classes, including high-molecular-weight PEGs, organic solvents and salts, coupled with pH (Majeed *et al.*, 2003 ▶).

**Figure 4:**
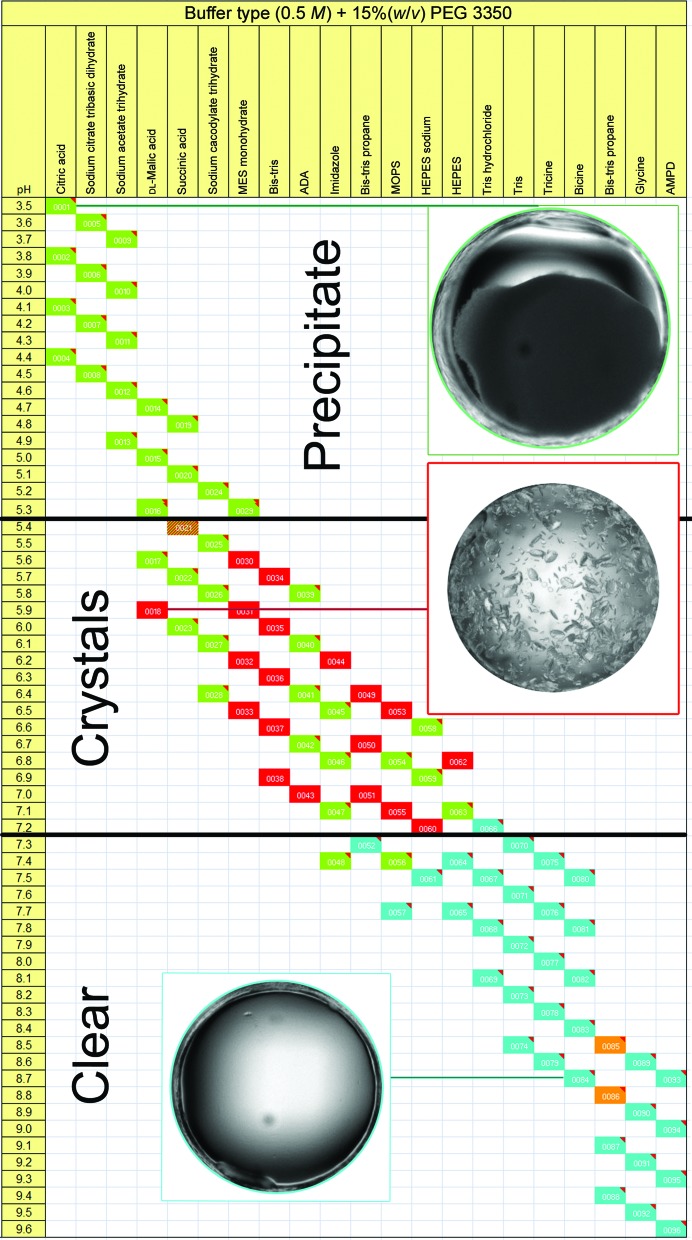
Chemical space layout of a pH/buffer-type screen. This clearly illustrates cases where having an identical chemical buffer at different pH and *vice versa* can alter the outcome of an experiment. Analysis of a putative glutathione-dependent formaldehyde-activating enzyme, pI = 6.88, with the Hampton Research Slice pH screen modified for microbatch with the addition of 15%(*w*/*v*) PEG 3350 and buffer concentrations of 0.5 *M*. Acidic pH produced heavy precipitate (green) in the range 3.5 ≤ pH ≤ 5.3. In the pH range 5.4 ≤ pH ≤ 7.2 crystals (red) or precipitates (green) formed depending on the pH and the chemistry. Mainly clear drops (blue) were formed in the range 7.3 ≤ pH ≤ 9.6. This screen very effectively distinguishes buffer pH from buffer-type effects on crystallization. The diameter of the circle is 0.9 mm.

Screening of pH at fine granularity (micro-pH increments) has been successfully used for the optimization of challenging protein crystals (McPherson, 1995 ▶); built upon this principle, and decoupling buffer chemistry from pH, the pH Slice screen (Hampton Research, Aliso Viejo, California, USA) samples pH in 0.1 pH-unit increments in the range 3.5 ≤ pH ≤ 9.6 using 20 chemically distinct buffers to determine pH *versus* buffer-type chemical effects. The results from pH Slice can readily be interpreted by arranging the cocktails as shown in Fig. 4 ▶.

### 4.6. Data mining to develop screens   

One of the results of the development of crystallization kits was the recognition that ‘high-throughput’ structural biology (more familiarly called ‘structural genomics’) was now a realistic scientific and technical goal. Recall that when structural genomics was first being considered, the vast majority of crystallizers were setting up vapour-diffusion experiments in 24-well plates by hand. The focus of structural genomics programs has evolved over time, but significant financial investment from both private and public sectors was directed into the creation of high-throughput experimental platforms for structural biology, and one of the aims of all of the projects was to collect sufficient information about the process, including crystallization, to develop a self-evolving, data-rich learning environment to improve methods rationally. As a result, all of the high-throughput crystallography platforms have amassed information, which has been used to guide the generation of yet more screens. The major difference between these screens and earlier data-mining efforts was that the structural genomics analyses include information about what went into crystallization as well as information about the successful (crystal-forming) and unsuccessful (crystals did not form) outcomes. One of the questions that can be asked, given both the initial screening information and the successful conditions, is ‘What is the smallest number of initial trials that would have given a similar overall result?’ Results from a structural genomics-style project on 755 nonmembrane proteins from six bacterial species, where each protein had been trialled in the (48-condition) Hampton Research Crystal Screen, showed that 45% of the samples showed some sign of crystallizing. Further analysis indicated that just six of the 48 conditions from this screen would have crystallized almost 60% of the proteins and that trialling the proteins against 24 conditions would have produced 94% of the total crystal hits (Kimber *et al.*, 2003 ▶). A similar analysis performed on *Thermatoga maritima* proteins at the Joint Center for Structural Genomics (JCSG) which had been set up in 480 initial conditions resulted in a set of 67 conditions which would have produced the bulk of the crystal hits (Page & Stevens, 2004 ▶). Perhaps one of the more interesting incidental observations from the JCSG study was that the 67 conditions contained a duplicate, and that different proteins showed different behaviours in the two (identical) conditions, clearly demonstrating the stochastic nature of the crystallization process. One of the outcomes of the early structural genomics projects, which mainly used the commercial screens, was that the PEG/Ion screen, produced by Hampton Research, was particularly effective at crystallizing proteins. The PEG/Ion screen is a very simple 48-condition screen where each condition contains 20%(*w*/*v*) PEG 3350 with the addition of a 0.2 *M* concentration of one of 48 different salts. Of course, generating one hit in a screen does not necessarily mean that the hit will be the only chemistry that will lead to successful structural studies; the recent success (and popularity) of matrix seeding (see below) attests to this.

Significant work remains to be performed from the perspective of data mining. The collection of vast amounts of data has been performed very successfully; however, communicating these data amongst centres and interpreting the results from large volumes of data remains challenging (Newman *et al.*, 2012 ▶).

### 4.7. Combination screens   

Researchers at the NKI Institute outside Amsterdam were struggling with the cost of crystallization and decided to implement a standard protocol that was limited in scope but that would be successful at both crystallizing proteins and providing further information about the protein sample if it did not crystallize (Newman *et al.*, 2005 ▶). This would have to be a combination of grids and sparse-matrix screening, and the result was two 96-condition kits, one based on the most successful cocktails identified by the Joint Center for Structural Genomics (JCSG) work and the other based on the known success of the PEG/Ion screen. The JCSG+ screen takes the 66 distinct cocktails from the JCSG set and adds 30 conditions from the commercially available Index screen, ensuring that the extra 30 cocktails were diverse in chemical composition and had a pH range to complement the range of the 66 conditions. The 96-cocktail pH, anion and cation-testing (PACT) screen consists of three individual PEG-based grid screens which test a protein’s response to a pH, cations and anions. The PACT screen can be subdivided into a 24-cocktail PEG/pH screen covering the range 4 ≤ pH ≤ 9 (using four multi-component buffer systems to decouple buffer chemistry from pH; Newman, 2004 ▶), a 24-cocktail cation/PEG screen and a 48-cocktail anion/PEG screen.

### 4.8. Not all screens are created equal   

From 1991, with the advent of the Jancarik and Kim screen and the first commercial instance of this screen, there has been an explosion in screens and other crystallization paraphernalia; today, well over 200 screens are commercially available. Some screens were placed on the market and did not last: what had seemed to be a good idea at the time turned out to have unforeseen problems. An example of this would be the OZMA screens, which were screens formulated with heavy metals, with the idea being that any crystal grown in these screens would be ‘auto-derivatized’ ready for extracting phase information. The downfall of these screens was that the metals rarely bound specifically enough to be used for phasing, but contributed enormously to the absorption of X-rays and thus to radiation damage during X-ray data collection. Other screens that seemed like a great idea, for example kinase-specific screens and nuclear hormone receptor screens, were too specialized and generally did no better than the general standard sparse-matrix screens. Initial screens with many factors in each condition make the tacit assumption that a factor that is not necessary for crystallization will be benign or neutral. Even if this is true, having many components complicates any required downstream optimization in two ways. Firstly, managing the design of the subsequent experiments in order to unambiguously tease out the contribution of each factor becomes more difficult, but also the optimization can be challenging when the chemicals in the screens are not readily available in the home laboratory, and the more factors in an initial condition the more likely this is to be the case

Duplication of screens amongst many vendors, essentially offering chemically identical screens by another name, is something to be aware of prior to committing protein, time and effort towards screening. Crystal Screen HT is a 96-condition screen extending the functionality of the original Crystal Screen sold by Hampton Research. Very similar screens can be obtained from Molecular Dimensions (Structure Screen I + II), Jena Bioscience (JBScreen Basic HTS), Qiagen (The Classics Suite) and Sigma (HT Kit). Adding to the confusion, not all of these screens will use the same chemical nomenclature and not all the cocktails will be listed in the same order. There is a webtool (http://c6.csiro.au) available to help identify chemically similar screens using a dictionary of standard chemical names and a distance metric to find similarities (Newman *et al.*, 2010 ▶).

### 4.9. Optimization   

In some cases the initial crystallization-screening experiment may produce a crystal that can be directly used to yield a model of the structure. However, more typically the production of X-ray-quality crystals occurs *via* optimization (Newman *et al.*, 2013 ▶). Optimization makes use of the information obtained from initial screening to develop strategies and crystallization cocktails which focus more narrowly on areas of crystallization space that are likely to produce crystals. Sophisticated strategies are available to design optimization experiments (see, for example, Carter & Yin, 1994 ▶; Carter & Carter, 1979 ▶; Carter, 1997 ▶; Shieh *et al.*, 1995 ▶). Other approaches are experimental and very suitable for application in a high-throughput setting (Luft *et al.*, 2007 ▶). Despite the recognition of the importance of optimization, there are no standard approaches. All, initially at least, vary the initial physicochemical conditions that produced the crystallization hit. Most often key variables will include the concentration of the chemical factors in the initial hit(s) or the pH (particularly for the components considered to be ‘buffers’); variables such as temperature can also be applied to great effect. Oddly, varying both the pH and the concentration of the buffer is rarely seen. The goal is to identify conditions that produce crystals that provide the necessary structural information to address the question being asked. In some cases this goal may be to produce large crystals, *e.g.* for neutron diffraction, in which case the optimization process is relatively straightforward as volume can be used as a quantitative parameter for a mathematical approach (Snell *et al.*, 2006 ▶). Unfortunately, in the case of X-ray diffraction studies the external appearance of the crystal often does not correlate to its diffraction properties, meaning that while crystal appearance can be used in a qualitative fashion (to find single crystals or crystals with sufficient volume for diffraction experiments), X-ray diffraction techniques are required to provide a quantitative metric against which to optimize. Whatever the approach, there are a number of guiding principles. (i) The same chemical approaches used for screening are used for optimization, but there are solubility limits and optimization must take place within these limits. (ii) Some chemicals have a lifetime, *e.g.* acidification of a PEG solution with time, temperature and light (Cudney, 2012 ▶), and when possible the same stocks should be used for optimization as have been used for screening. (iii) Some chemicals in commercial kits are expensive or difficult to get hold of on their own. (iv) Protein preparations can vary: always try and preserve some of the identical preparation for the optimization step. (v) Replication pays off: crystallization is a stochastic process and if you have enough protein it is worth replicating the optimization experiments (Newman *et al.*, 2007 ▶).

**Figure 5:**
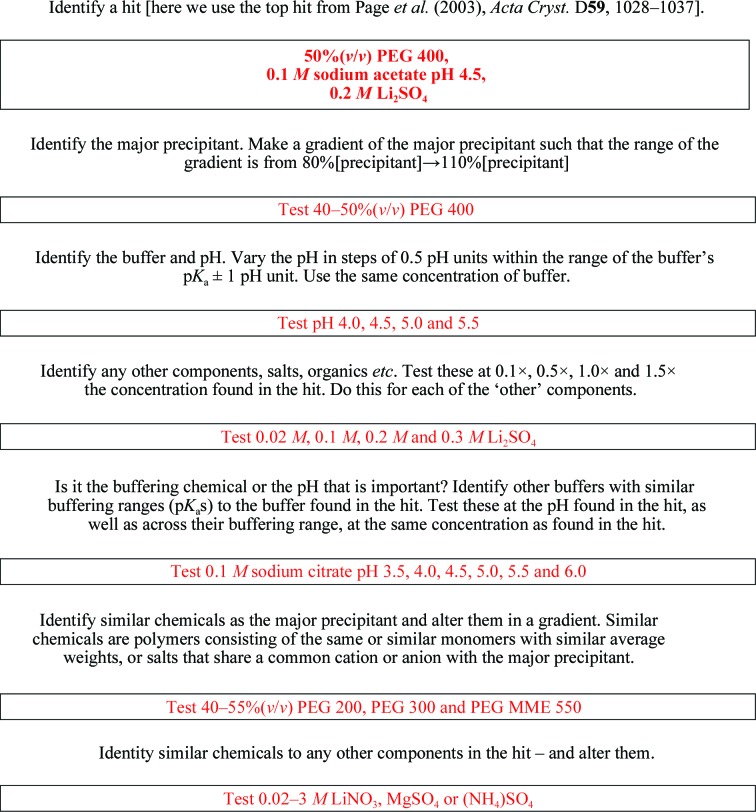
Optimization flowchart. This flowchart illustrates the case described in the text where an initial crystallization condition of 50%(*v*/*v*) PEG 400, 0.2 *M* lithium sulfate, 0.1 *M* sodium acetate pH 4.5 is used as a starting point to optimize crystals, presumably for diffraction analysis.

To practically expand on the general comments about optimization, it is useful to take an example of the screening process and how the information and knowledge of the components of the screens drives subsequent steps (Fig. 5 ▶). The top-performing cocktail in a shotgun strategy approach to structural genomics targets was a crystallization condition consisting of 50%(*w*/*v*) PEG 400, 0.1 *M* sodium acetate, 0.2 *M* lithium sulfate (Page *et al.*, 2003 ▶). If an initial hit resulted from this cocktail, we would start from this hit and explore the surrounding conditions guided by other results. We can make use of the experimental design methods described above, but for the sake of simplicity we will consider optimization around two dimensions. The major precipitant is the polymer PEG 400 and (beyond the ratio of protein and precipitant discussed below) we have two other variables: the buffer, sodium acetate, and the salt, lithium sulfate. The buffer pH has a major influence on crystallization outcome and because of this we would choose this as the second variable to optimize. In a fine screen with many conditions we may already have knowledge about the influence of these variables and this would guide our sampling strategy; similarly, we also have knowledge about solubility and whether it is possible to make a selected chemical cocktail beyond the concentration range used for screening. Finally, based upon the p*K*_a_, we know the effective buffering range of the buffer used. This knowledge guides the optimization approach. For a screen that samples chemical space with lower fidelity, we would start by constructing two chemical gradients, in the case of PEG a range from 80 to 110% of the initial concentration. The effects of PEG on protein solubility are nonideal and nonlinear. PEG has been described as

an inert solvent sponge that indiscriminately raises the effective concentration of all the proteins, those of larger size being somewhat more sensitive than smaller one

(Atha & Ingham, 1981 ▶). The buffer, sodium acetate, has an effective pH range of 3.7–5.6, so we might explore pH 4.0–5.5 in steps of 0.5 pH units, keeping the value of the buffer concentration identical to the initial hit. In this case lithium sulfate is also present, but we may not know how this (or other components, salts, organics *etc.*) influences the outcome. We would replicate the optimization with each of these components at 0.1, 0.5, 1.0 and 1.5 times the initial concentration. It quickly becomes apparent why experimental design approaches need to be considered. At this point the results describe the response of the protein to a highly defined area of chemical space. To further tweak this response and to obtain the best quality crystals, the next steps could be to explore other buffer types with an effective buffering range that includes the original hit but extends the pH range beyond it. For example, in this case sodium citrate has a buffering range from pH 3.0 to 6.2 and we could explore the influence of chemical buffer type and pH range by utilizing sodium acetate buffer to determine whether we can replicate the original citrate hits while simultaneously determining whether extending the pH range is an effective optimization strategy. We would also look at similar precipitants. In this case PEG 400 is similar to PEG 200, PEG 600 or PEG MME 550. A more distant chemical relationship would be MPD, which can often be used in place of low-molecular-weight (liquid) PEGS. Similarly, the lithium sulfate could be substituted by similar salts, for example lithium chloride, magnesium sulfate or sodium sulfate.

While this paper and the example above focus on the chemical screens, other parameters have an influence, for example the ratio of components, the temperature or the crystallization method. Using the microbatch method, simply varying the ratio of the protein to the cocktail and probing temperature is a powerful optimization strategy (Luft *et al.*, 2007 ▶). In vapour- or liquid-diffusion methods, the kinetics of equilibration can be varied to great effect (Luft & DeTitta, 1997 ▶). Even the crystallization geometry (Luft *et al.*, 1996 ▶) and drop volume (Fox & Karplus, 1993 ▶) can significantly influence the outcome.

Another approach is to use additives. A ‘base condition’ containing the reservoir from the best hit can be used with a small amount, *e.g.* 10%, of something else, for example a commercial additive screen or even other crystallization-screen components.

Seeding approaches can be particularly effective to increase the number of cocktails producing hits from a crystallization screen; techniques such as microseed matrix screening (D’Arcy *et al.*, 2007 ▶), where microseeds are introduced during the setup of an initial crystallization screen, can dramatically increase the number of lead conditions. Seeding is an extremely effective tool for crystal volume optimization, where even liquid–liquid phase separation or precipitates can be used as a seed stock to produce larger volume crystals (Bergfors, 2003 ▶).

The screening and optimization processes are linked by the chemistry and the dynamics of the crystallization process. While experience breeds knowledge, this experience is not required to set up a commercial crystallization screen. This can lead to difficulties for a novice when large single crystals do not result from the initial screen. Optimization has a vast number of variables and requires some foreknowledge, consideration and thought for the experimental design. From the experimental perspective, optimization is less straightforward than initial screening.

## 5. Storing crystallization knowledge   

Many of the common crystallization screens today were designed around crystallization knowledge. The BMCD, initiated in 1989, played an important role in this by being a repository of this knowledge (Gilliland, 1988 ▶; Gilliland *et al.*, 1994 ▶; Tung & Gallagher, 2009 ▶). The BMCD is available online and is one of the earliest Standard Reference Databases at NIST. When the first version of the BMCD was deployed, access was achieved only after receiving a floppy disk of the database. The original version of the BMCD precedes internet-enabled rapid access to crystallization data; it was developed through tremendous and meticulous efforts to review and compile crystallization data from the literature, one protein at a time. Often the data were incomplete, making the task incredibly challenging. The current version (4.03) of the BMCD contains standardized crystallization data for 43 406 crystal entries which have been extracted from PDB REMARK 280 records. The data in PDB REMARK 280 is not standardized; it requires significant effort to obtain information about crystallization trends from this data (Peat *et al.*, 2005 ▶).

The BMCD enabled cluster analysis to identify chemical trends in crystallization behaviour based upon the class of the macromolecule (Samudzi *et al.*, 1992 ▶). It also led to the development of software to design crystallization screens that were not weighted equally from a chemical perspective; chemicals could be weighted according to their success at crystallizing proteins in a similar hierarchal classification (Hennessy *et al.*, 2000 ▶). While the BMCD is a tremendous resource, it is important to recognize that the data are limited to the chemical conditions that produced the crystal used to determine the crystallographic structure. Therefore, we do not know whether a protein is incapable of crystallizing from another chemical condition, whether it was never tested or whether it crystallizes but simply was not structurally pursued.

The data generated by worldwide structural genomics efforts is much more comprehensive in this regard. Structural genomics approaches are systematic; that is, crystallization screening uses standardized protocols. Based on data mining of targets from a structural genomics centre, investigators identified a set of protein properties that could be calculated from the primary sequence and used to classify a protein into one of five crystallization classes ranging from very difficult to optimal (Slabinski, Jaroszewski, Rodrigues *et al.*, 2007 ▶; Slabinski, Jaroszewski, Rychlewski *et al.*, 2007 ▶). A separate study of structural genomics targets found that crystallization propensity is correlated with well ordered surface epitopes that can promote intermolecular interactions and developed an approach to predict the probability of determining a crystallographic structure from the primary sequence based on this data (Price *et al.*, 2009 ▶). Unfortunately, there is not a standard format for crystallization data, making it a challenge to attempt inter-centre investigations (Newman *et al.*, 2012 ▶).

## 6. Screening experiments are limited by vision   

A crystallization screen is only as good as our ability to observe the outcomes. Taken to the extreme, even if every experiment produces a protein crystal the result is of little consequence if it goes undetected by the investigator. When we use an assay and are fortunate enough to hit upon chemical conditions that produce obvious crystals that are large enough to easily recognize under a microscope or in an image of the experiment, it is easy to recognize success. At this point, we can characterize the crystal to make certain that it is crystalline and proteinaceous and test the quality of X-ray diffraction. However, crystallization screens are based upon sampling of chemical space, and more often than not the chemicals being sampled will not provide obvious crystals but other types of outcomes. These outcomes range from clear drops to heavy precipitate, and can include protein skin, phase separation, dust, fibres and even the (very) occasional insect. The interpretation is the crux: for example, it may be crucial to distinguish between a ‘good’ heavy precipitate (one where the protein remains well folded but has come out of solution) and the situation where the protein has denatured under the environment engendered by the cocktail and has undergone amorphous aggregation. In particular, clear drops can be difficult to interpret, as they look identical to the eye and yet can fall in a thermodynamic range from undersaturated to metastable supersaturation. At metastable supersaturation, crystallization is thermodynamically, but not kinetically, favoured; these conditions are incredibly close to crystallizing the protein and could produce a crystal through an event that increases the level of supersaturation, which could include further dehydration of the drop, a change in temperature or the purposeful or accidental addition of a nucleant. Other metastable outcomes, such as liquid–liquid phase separation, can again be very close to crystallization and may only require a change in temperature to trigger a nucleation event (Broide *et al.*, 1996 ▶). Precipitate can be amorphous or microcrystalline. These microcrystalline precipitates are often missed by investigators because the tools required to identify them as microcrystals are not applied or are unavailable. Birefringence can help an investigator to distinguish microcrystals from an amorphous precipitate (Echalier *et al.*, 2004 ▶), as many crystals will show colour when viewed with cross-polarisers. It is important to note that using cross-polarisers allows one to potentially distinguish crystals from other outcomes, but does not allow differentiation between salt crystals and protein crystals. Most crystallization experiments will display more than one ‘result’: combinations of crystals and precipitate, skin and precipitate, or denatured protein and phase separation are often observed in the same experimental drop (Luft, Wolfley *et al.*, 2011 ▶). An article in this series on the visualization of crystals will address this topic.

## 7. Analyzing the process of crystallogenesis   

It is unfortunately naïve to expect that any given protein sample, when set up in one or more commercial screens, will produce crystals, let alone diffraction-worthy crystals. A recent analysis of crystallization papers published in *Acta Crystallographica Section F* showed that 75% of the systems required some optimization (Newman *et al.*, 2013 ▶), and most of the papers reported the crystallization of fairly simple macromolecules: soluble, single proteins from bacterial systems. The skill in crystallogenesis lies not in identifying large crystals (which is easy, but still extremely gratifying) but in finding those conditions which are close to producing large crystals. This explains our reliance on the phase diagram. The phase diagram suggests that the area in which crystals will grow will be intermediate between the area where the protein is undersaturated (clear drops) and the areas where the protein has come out of solution as a precipitate, either amorphous or microcrystalline. After the initial screens are dispensed, we analyse the results in terms of looking for trends: in effect, building up phase diagrams for different chemicals. This is performed explicitly in the analysis package *AutoSherlock* (Snell *et al.*, 2008 ▶), but we must remember that the interpretation of such a phase diagram in multiple dimensions is often not an easy task owing to the extreme sparseness of the sampling of chemical space.

There is a further point to consider: the aim of an X-ray structure is overwhelmingly to understand a biological system, and we then have further restraints on how the crystals may be grown. The requirement that the protein be in a form which is appropriate for crystallographic analysis may lead to chemical restrictions, such as the pH range or general chemical environment, that are not compatible with the desired biochemical analysis of the functional mechanisms of the protein. Another common requirement is the production of suitable crystals for subsequent small-molecule interaction studies. The small molecules are most conveniently diffused into an existing protein crystal, but this requires the production of crystals which have accessible active sites and suitable growth conditions for ligand compatibility. In these cases seeding from one crystal form into an initial screen (matrix seeding) can produce crystals of different habit and packing, grown under different conditions, if initial crystals were obtained but were unsuitable for the purpose at hand (Obmolova *et al.*, 2010 ▶; Ireton & Stoddard, 2004 ▶; D’Arcy *et al.*, 2007 ▶; Newman *et al.*, 2011 ▶).

## 8. Stochastic events and dumb luck   

It must be kept in mind that crystallization events are stochastic: the experimental results are not 100% reproducible. There is evidence that setting up replicate experiments, rather than additional cocktails, may be a path to success (Newman *et al.*, 2007 ▶). A cocktail that has conditions where the protein is labile, with a high enough level of supersaturation for spontaneous, homogeneous nucleation to occur, will be more likely to be reproducible in subsequent experiments if the condition is at a higher level of supersaturation than if it sits very close to a metastable boundary. Because crystallization screens are generally designed to have significant variation in their chemical composition, as generic screens will be used for proteins having many different chemical and physical characteristics, there are cocktails where one protein may completely precipitate from solution while another will remain a clear drop. Clear drops can be deceiving as they could indicate undersaturation, saturation or a metastable condition that from a thermodynamic perspective will crystallize, but kinetically nucleation is not probable. Stochastically, a single nucleation event is less likely to occur than hundreds of nucleation events. Replication of an experiment that produces only one crystal could very readily result in a clear drop and replication of a clear drop in this region could well result in a crystal. Even better is replication with seeding; as it is known that the nucleation step is random, with a supersaturation-dependent frequency, adding nucleation sites can induce crystal growth where none was seen before. There is an extensive literature on the use of seeding: from its use as an optimization tool (Bergfors, 2003 ▶) to its use in reliably obtaining crystals for fragment screening (Newman *et al.*, 2009 ▶) and, most recently, its use in obtaining initial leads, so-called ‘matrix seeding’ (D’Arcy *et al.*, 2007 ▶; Villaseñor *et al.*, 2010 ▶). Furthermore, in parallel with the expansion of seeding as an adjunct to screening has been the development of techniques for screening using the current crystallization dispensing technology (Villaseñor *et al.*, 2010 ▶; Newman *et al.*, 2008 ▶).

The stochastic nature is compounded by the human variable that often hides in plain sight. Anecdotal evidence would suggest that some investigators are far more successful with crystallization than others. With the nature of the target put aside, the likelihood of being in the former category increases with attention to detail. Oftentimes, a specific, critical variable is not recognized beforehand. These variables are easily missed and altered without our knowledge; they can include temperature changes, unintentional chemical variations [contaminants, or the aging of a PEG solution (Cudney, 2012 ▶)] and inadvertent differences in samples or sample-handling protocols. Collectively, these variables have been described, and appropriately referred to as ‘dumb luck’ (Cudney, 1999 ▶). Good laboratory practices play as important a role in obtaining and optimizing the crystal as the screens used for crystallization. Automation is not a substitute for attention to detail, recording of all relevant data and thoughtful analysis of the results.

## 9. How has crystallization history influenced practice?   

Rather than create a discussion section to address the title of our paper, we leave it to the reader to decide whether crystallization history has had a positive, a negative or a mixed impact on the research efforts in this field. Most researchers use structural biology as a tool to provide insight about the biological system that they are studying, and as long as they are reasonably successful their focus is not on the science behind crystallogenesis. It is inconceivable to those that study crystallization as a science itself that one would be exuberant when one observed crystals in a condition containing ammonium sulfate if the protein sample itself contained calcium. Similarly, one would try to avoid a phosphate buffer to formulate their protein sample. Like anything else, familiarity breeds knowledge; crystallization kits, because of their convenience, have opened up the field of crystallogenesis, and as yet there are few tools available to provide a contrapuntal expert knowledge background. What other collective knowledge is missing: what is the buffering range of any given buffer? How far away from a p*K*_a_ might one stray? What is the solubility of many of the common salts? Why do PEGs become acidic in sunlight and heat (Cudney, 2012 ▶)? Consider the rationale for having PEG 3K, 3350 and 4K in our crystallization laboratories. PEG 3350 is an FDA-approved polymer, with a narrow distribution of molecular weights, which is why it is used in our crystallization screens. However, even though (or maybe because) PEG 3350 has FDA approval, it contains small and reproducible amounts of phosphate, such that a 30%(*w*/*v*) PEG 3350 solution will contain ∼1 m*M* phosphate. At high concentrations of PEG 3350, divalent cations can produce nearly insoluble phosphate salts which can and frequently do fool a jubilant but unknowing victim into thinking they have crystallized their protein. It is always easier in the short term to just set up the experiments, but making oneself aware of the prior art will almost certainly save time in the long term; although it delays the instant gratification of setting up the experiments, it will be more likely to provide a more meaningful instant gratification upon seeing actual protein crystals.

To some extent, the past very much influences the future: for example, why do we see sodium acetate buffer at pH 4.6? Sodium acetate has a p*K*_a_ of 4.76, so logically the most profound buffering will be when the pH of an acetate solution is between pH 4.7 and 4.8. Mankind’s pleasure in ‘round’ numbers might suggest that pH 4.5 or pH 5.0 would be appropriate pH points for acetate. But pH 4.6 is the solubility maximum for lysozyme, where the largest crystals were obtained (Ataka & Tanaka, 1986 ▶), and this result was first obtained using an acetate buffer and has been captured for posterity in a number of modern screens. The studies by the structural genomics centres which tested the Crystal Screen of Jancarik and Kim against large numbers of proteins never picked the yellow, ferric chloride-containing condition as being a particularly useful cocktail, and yet ferric chloride was obviously found often enough at the time that the screen was developed to have made the cut into that first set of 50 cocktails. In the 1980s, many of the existing structures would have been globins and other haem-containing proteins, and the iron may well have been found in a number of these crystallization conditions, included perhaps to help stabilize the haem.

Another example is the preponderance of only two common temperatures. Temperature is a generally applicable variable that directly affects solubility and therefore crystallization; in one study 24 out of 28 proteins had a temperature-dependent solubility (Christopher *et al.*, 1998 ▶). Protein solubility is dependent on the solvent conditions and can be directly or inversely related to temperature based upon the solvent (Luft *et al.*, 2007 ▶). The potential of using temperature for automated setups has long been recognized (Chayen *et al.*, 1990 ▶). It is unfortunately the case that temperature, as a variable, suffers from extreme oversampling at two values, as shown by the data in the BCMD. It is often the case that a laboratory will only have access to temperatures of 277 K (a refrigerator, cold room or incubator) and room temperature, but little else. It is rarely the case that temperature is optimized in finer gradations to identify the best temperature for the crystallization of a particular protein (Luft *et al.*, 1999 ▶).

While we can sample many kits to try crystallization, it is worth noting that many of the original developments that enabled these kits came from fields outside of crystallization research. The history of crystallization screening is tied directly to the history of protein fractionation and purification. Chemicals used to fractionate and isolate a single purified protein from mixtures of proteins are the source and rationale for the inclusion of many of the chemicals found in contemporary crystallization screens. The addition of neutral salts for protein separation has obvious ties to modern-day protein crystallization. A monograph written by Prosper Sylvain Dénis in 1856 states that salting-out is the only generally applicable method for the separation of proteins (Denis, 1856 ▶). The separation and purification of ‘proteids’ by crystallization was considered by Samuel Barnett Schryver to be a major breakthrough:

… the elaboration of methods for the crystallization of certain substances of this class must be considered as a distinct advance in the chemical technique for the preparation of pure substances.

(Schryver, 1913 ▶). Temperature, pH adjustments and fractionation by salts were the three major technologies employed to purify and then crystallize proteins during the early to mid 20th century.

Another example of a protein-purification technology is the use of tags to aid purification. Initially, tags were generally small peptides that could only be recognized by very specific antibodies: the production of those monoclonal antibodies could escalate the cost of the capture columns beyond the reach of most laboratories. The introduction of cheap, universal capture systems (GST, His tags) forever changed purification in the late 1980s. The idea of a universal tag was very successfully applied in the crystallization of G-coupled protein receptors (GPCRs): the choice of T4 lysozyme was inspired, as the formidable body of work on this protein in the laboratory of Brian Matthews has shown that every point on the surface of the protein could make a crystal contact (Baase *et al.*, 2010 ▶).

One of the most successful crystallization agents, PEG, has its origins in protein fractionation. Several high-molecular-weight linear polymers, including polyethylene glycol, dextran, nonylphenol ethoxylate, polyvinyl alcohol and polyvinyl pyrrolidone, were studied for their effectiveness at selective fractionation as a means to isolate highly purified proteins from the blood (Polson *et al.*, 1964 ▶). The group reported

Polyethylene glycol (mol.wt. 6000) appears to be the most suitable protein precipitants in this group because its solutions are less viscous and cause virtually no denaturation at room temperature.

It is interesting to consider that we could be using starches such as dextran for crystallization if this study had gone differently. The first protein crystallized using PEG was alcohol dehydrogenase (Janssen & Ruelius, 1968 ▶). The first systematic evaluation of PEG as a crystallization reagent was undertaken by McPherson (1976*a* ▶), who based on his study of 22 proteins, where 13 out of 22 crystallized from a screen of four concentrations of five PEGs (400, 1K, 4K, 6K and 20K), and concluded that

if one were to attempt the crystallization of a macromolecule which had never previously exhibited crystallinity, or for which only a very small amount of material was available for the trials, a judicious initial choice for the screening would be PEG.

## 10. The future   

The vast majority of today’s practitioners of protein crystallization are using crystals as a tool to achieve a structural goal; the scientific exploration of crystallization is not their primary or even secondary objective. Crystallization with modern-day screens is just successful enough, with approximately 20% of samples yielding a structure, that the detailed study of the process and how to improve it is of a lower priority than if these screens had been less successful. The crystallization problem remains far from solved, yet emphasis on and financial investment in this research has certainly declined from its peak during the 1990s. This paper has focused solely on the formulation and crystallization screening of soluble proteins, ignoring the more challenging topics of complex, glycoprotein and membrane-protein crystallization. We do not have a good understanding of macromolecular crystallization; hence, the approach the field has devised is an empirical approach to resolve the problem. Crystals are critical for structural biology; structural biology is critical for biomedical discovery, agriculture and many other fields of research. Focused scientific investigations will be required to fully comprehend the complicated process of protein crystallization. It is unlikely that we will find the answers through data-mining efforts or computer simulations as the questions are too numerous and our understanding too poor. Will nanocrystallography, an event horizon, make the study of crystallization passé? This is unlikely, because even nanocrystallography (with its own unique problems) requires crystals, and the approach to this problem, the search and the screening are all based upon finding a needle in a chemical haystack. It is not a question of whether or not the crystallization problem can be solved, so much as a question of who will invest the financial resources and research efforts to finally truly understand this critically important and poorly understood process.

In summary, we would contend that crystallization history has had a mixed impact on practice, greatly enabling the technique through a plethora of different crystallization screening kits and hardware but at the same time masking some of the thought that could be applied, especially in more recalcitrant cases.
